# The Effect of Foliar Selenium (Se) Treatment on Growth, Photosynthesis, and Oxidative-Nitrosative Signalling of *Stevia rebaudiana* Leaves

**DOI:** 10.3390/antiox10010072

**Published:** 2021-01-08

**Authors:** Péter Borbély, Árpád Molnár, Emil Valyon, Attila Ördög, Klára Horváth-Boros, Dezső Csupor, Attila Fehér, Zsuzsanna Kolbert

**Affiliations:** 1 Department of Plant Biology, University of Szeged, Közép Fasor 52, H-6726 Szeged, Hungary; borbely.peter@atk.hu (P.B.); molnara@bio.u-szeged.hu (Á.M.); emil6555@citromail.hu (E.V.); aordog@bio.u-szeged.hu (A.Ö.); feher.attila@brc.hu (A.F.); 2 Department of Plant Molecular Biology, Agricultural Institute, Centre for Agricultural Research, Brunszvik u. 2, H-2462 Martonvásár, Hungary; 3 Department of Pharmacognosy, Faculty of Pharmacy, University of Szeged, Eötvös u. 6, H-6720 Szeged, Hungary; boros.klara@pharmacognosy.hu (K.H.-B.); csupor.dezso@pharmacognosy.hu (D.C.); 4 Biological Research Centre, Eötvös Lóránd Research Network, Temesvári krt. 62, H-6726 Szeged, Hungary

**Keywords:** nitrosative signalling, oxidative signalling, photosynthesis, selenium, *Stevia rebaudiana*

## Abstract

Selenium (Se) enrichment of *Stevia rebaudiana* Bertoni can serve a dual purpose, on the one hand to increase plant biomass and stress tolerance and on the other hand to produce Se fortified plant-based food. Foliar Se spraying (0, 6, 8, 10 mg/L selenate, 14 days) of *Stevia* plantlets resulted in slightly decreased stevioside and rebaudioside A concentrations, and it also caused significant increment in stem elongation, leaf number, and Se content, suggesting that foliar Se supplementation can be used as a biofortifying approach. Furthermore, Se slightly limited photosynthetic CO_2_ assimilation (A_N_, g_sw_, C_i_/C_a_), but exerted no significant effect on chlorophyll, carotenoid contents and on parameters associated with photosystem II (PSII) activity (F_V_/F_M_, F_0_, Y(NO)), indicating that Se causes no photodamage in PSII. Further results indicate that Se is able to activate PSI-cyclic electron flow independent protection mechanisms of the photosynthetic apparatus of *Stevia* plants. The applied Se activated superoxide dismutase (SOD) isoenzymes (MnSOD1, FeSOD1, FeSOD2, Cu/ZnSOD1, Cu/ZnSOD2) and down-regulated NADPH oxidase suggesting the Se-induced limitation of superoxide anion levels and consequent oxidative signalling in *Stevia* leaves. Additionally, the decrease in S-nitrosoglutathione reductase protein abundance and the intensification of protein tyrosine nitration indicate Se-triggered nitrosative signalling. Collectively, these results suggest that Se supplementation alters *Stevia* shoot morphology without significantly affecting biomass yield and photosynthesis, but increasing Se content and performing antioxidant effects, which indicates that foliar application of Se may be a promising method in *Stevia* cultivation.

## 1. Introduction

*Stevia rebaudiana* (Bertoni) belonging to the *Asteraceae* family is a perennial herb, cultivated for its sweet leaves which have been used as a sweetener in South America for centuries and is consumed worldwide nowadays [1]. The *Stevia* leaf contains several steviol glycosides (SGs). The major representatives of these diterpenes are stevioside, rebaudioside A, rebaudioside C, and dulcoside A [2]. However, in total, more than 30 different SGs with varying concentrations have been identified in the Stevia leaf [3]. SGs are synthetized *via* the plastid localized methylerythritol 4-phosphate (MEP) pathway from the precursors glyceraldehyde 3-phosphate (G3P) and pyruvate [4,5]. Since G3P derives from photosynthesis and the MEP pathway also supports the synthesis of photosynthetic pigments (chlorophylls and carotenoids), SG formation is tightly associated with photosynthetic activity and some SGs might contribute to the protection of the photosynthetic apparatus against environmental stresses [6].

Due to its SG content, *Stevia* leaves are 100–300-fold sweeter than sucrose and have zero calories, zero carbohydrates and don’t increase blood sugar level [1]. Beyond SGs, high flavonoid and phenolic content provides antioxidant potential for *Stevia* leaves [7,8,9]. Additionally, *Stevia* leaf extract also exerts antifungal and antimicrobial activities [10,11]. Due to these advantageous properties, the therapeutic application of *Stevia* against type II diabetes, obesity, cancer, hypertension, oxidative stress, dental caries and microbial infections receives great attention [12]. Thus, current research efforts are also focused on increasing SG content of *Stevia* leaves or improving biomass production during cultivation.

Selenium (Se) is a metalloid being present in the environment and is taken up by plant roots as selenate (SeO_4_^2−^) and selenite (SeO_3_^2−^). Due to its chemical similarity to sulphur, selenate is absorbed and assimilated in association with sulphate uptake and assimilation [13,14], while phosphate transport system is involved in selenite uptake [15]. Contrary to animals and humans, Se is not essential for land plants but at low concentrations it is beneficial for plant development and stress tolerance. Selenium has been reported to induce seed germination, promote vegetative growth, regulate reproductive growth, and delay senescence in plants grown in stress-free environments. Additionally, several studies reported that Se treatment ameliorated the damages caused by abiotic stresses like salinity, drought, heavy metals, high and low temperature, high light or UV-B radiation (reviewed in [14]. The positive effect of Se on stress tolerance may be attributable to its ability to regulate osmotic potential and turgor inducing the accumulation of soluble sugars and free amino acids, and to increase the transpiration rate [16]. Additionally, Se proved to enhance proline, carotenoid, and chlorophyll contents of plants [17]. It also mitigates the production of reactive oxygen species (ROS), such as hydroxyl radical (^●^OH), hydrogen peroxide (H_2_O_2_), or superoxide anion radical (O_2_^●−^) [18], directly or indirectly through the activation of antioxidant enzymes (e.g., superoxide dismutase SOD, catalase, ascorbate peroxidase etc.) and non-enzymatic antioxidants (e.g., ascorbate, glutathione) (reviewed in [19]).

Beyond ROS, plant cells produce also reactive nitrogen species (RNS) like nitric oxide (NO), S-nitrosoglutathione (GSNO) and peroxynitrite (ONOO^−^), which transfer their bioactivity primarily through posttranslational modifications including *S*-nitrosation and tyrosine nitration [20]. The degree of *S*-nitrosation is regulated by GSNO reductase (GSNOR), which catalyses the conversion of GSNO to GSSG and NH_3_ and thus it moderates GSNO-dependent nitrosative signalling [21]. Protein tyrosine nitration is a posttranslational modification catalysed by ONOO^−^ that results in the alteration of protein structure, activity, protein-protein interactions or subcellular localization. Tyrosine nitration-triggered activity loss has been observed in some plant enzymes and the competition of nitration with tyrosine phosphorylation has also been suggested. [22] Selenium exposure has been shown to induce the production of NO, ONOO^−^ and GSNO mostly in non-accumulator/sensitive plant species [23,24,25,26,27,28,29]. As a consequence of Se-triggered RNS production, the intensification of protein tyrosine nitration has also been observed in Se sensitive species like pea or *Astragalus membranaceus* [25,28].

Based on the above-mentioned beneficial effects of Se, fertilization of crops with inorganic Se forms (selenate or selenite) has been applied and the impacts on plants have been extensively studied (reviewed in [22]). Foliar application of Se represents a highly efficient, economic, and environmentally friendly way of Se administration during plant cultivation [30]. Compared to soil Se application, foliar Se fertilization is up to eight times more efficient [31]. It has been reported by several authors, that the cuticle is permeable to both organic and inorganic ions and undissociated molecules and the penetration of ions is influenced by the charge, absorbability, and ion radius [32,33,34].

Se enrichment can serve a dual purpose, on the one hand to increase plant biomass and stress tolerance and on the other hand to produce Se fortified plant-based food, which can be a rich source of dietary Se. For humans, Se is essential and the recommended daily intake for adults has been determined as 55 μg/kg Se [35,36,37]. Insufficient Se supply, which is common in low Se containing soils such as agricultural soils in Hungary [38,39], contributes to diseases including cardiovascular disease, cognitive impairment, hypothyroidism, immune insufficiency, male fertility issues may develop [40].

Considering the beneficial health effects of both Se and *Stevia*, studying the effect of Se administration on *Stevia* plants is timely and relevant. Our knowledge regarding the details of the modifying effect of beneficial Se dosages on photosynthesis and on oxidative-nitrosative signalling is limited. Therefore, the goal of this research was to explore the effects of Se on shoot growth, photosynthesis and oxidative-nitrosative signalling in *Stevia* and to investigate the Se increasing potential of low-dose Se foliar spraying of *Stevia* plants.

## 2. Materials and Methods

### 2.1. Plant Cultivation and Se Treatment

Vegetatively propagated *Stevia rebaudiana* Bertoni plantlets with three fully-developed leaves were obtained from Bíró Horticulture and Trade Corp. (Szigetszentmiklós, Hungary). Plantlets were transplanted in 0.3 L plastic pots filled with conventional soil mixed with peat and were placed in a growth chamber (Aralab, Rio de Mouro, Portugal) under long days (16 h, 23 °C, 300 µE light intensity and 8 h, 18 °C darkness) with a relative humidity of 70%. Plants were grown for two weeks and watered three times a week. Sodium selenate (Na_2_SeO_4_) solutions (6, 8, or 10 mg/L) were prepared in distilled water and 0.1% TWEEN 20 was added to each solution. Concentrations were determined based on available literature and pilot experiments. The shoots of the plants were sprayed with distilled water or with selenate solutions (three sprayings per plant) and shoots were covered with transparent plastic foil to isolate the different treatments. After one week, the Se spraying was repeated, and following another week, in vivo measurements were performed or the leaf samples were taken in liquid N_2_ and stored at −80 °C until processing. For measurements (except growth parameters) only fully expanded, but non-senescing leaves (3rd leaf counted from the apex) were used.

### 2.2. Measurement of Growth Parameters

The total number of leaves was counted manually and expressed as number/plant, stem height (cm) was measured using a scale, while leaf and stem fresh weight (g) were measured using a balance. Dry weight of the leaves and stems were determined after 72 h of drying at 70 °C.

### 2.3. Measurement of Se Content

Leaf material of *Stevia* was extensively washed in distilled water then dried at 70 °C for 72 h. 4 mL of nitric acid (69%, *w*/*v*) and 2 mL of hydrogen peroxide (30%, *w*/*v*) were added to 100 mg of dried leaves which was followed by the microwave-assisted destruction of samples at 200 °C and 1600 W for 15 min (Mars Xpress 5, CEM Corp., Matthews, NC, USA). After digestion samples were diluted with ddH_2_O to the final volume of 20 mL. Se concentrations were determined by inductively coupled plasma mass spectrometry (ICP-MS) (Agilent 7700 Series, Santa Clara, CA, USA) using ^89^Y as internal standard. Se concentrations are given in µg/g dry weight (DW).

### 2.4. Measurement of Stevioside and Rebaudioside A Contents

*Stevia* leaf samples were dried at room temperature and chopped in a Retsch SM 100 chopper at 8000 rpm for 20 s. 100 mg of the samples were extracted using an ultrasonic bath at 30 °C for 10 min with 10 mL of a mixture of methanol-water 1:1 and then filtered through a PTFE syringe filter. All samples were extracted in triplicate. The steviol glycoside content of the samples was analysed using a HPLC system comprising a Shimadzu LC 20AD pump, DGU 20A5R degasser, SIL20ACH autosampler (tempered to 25 °C), CTO-20AC column oven, and SPD-M20A photodiode array detector modules, connected with CBM-20A control module, on a Kinetex^®^ 2.6 µm XB-C18 100 Å (150 × 4.6 mm) column. Column temperature was set to 70 °C. Isocratic elution was carried out with the mixture (68:32) of 0.1% trifluoro-acetic acid in water (A) and MeCN (B). Gradient elution was applied (eluent B 20-40-55-70-70-20-20% in 1-20-24-27-28-28.5-33-34 min). The flow rate was 1.5 mL/min. Detection was carried out in the whole UV wavelength range, and specifically at 205 nm. All extracts were analysed in triplicate.

### 2.5. Measurement of Pigment Concentrations

Measurement of pigment content was performed according to Lichtenthaler [41] with slight modifications. 25 mg of plant leaf tissue was homogenized and incubated with 1 mL of 100 (*v*/*v*) % acetone at 4 °C for 24 h. The sample was centrifuged at 9300× *g*, 4 °C for 10 min, the supernatant transferred to an Eppendorf tube and 1 mL of 80 (*v*/*v*) % acetone was added to the plant material. After 24 h of incubation, the plant material was centrifuged again, the two supernatants were united and measured. Pigment concentrations were calculated from the data according to Lichtenthaler [41] and presented as µg/mL.

### 2.6. Measurement of Chlorophyll a Fluorescence and PSI Activity

The simultaneous measurement of chlorophyll *a* (Chl *a*) fluorescence and PSI absorbance changes were carried out with a Dual-PAM-100 fluorometer (Heinz Walz GmbH, Effeltrich, Germany). In dark adapted leaves, the minimal fluorescence level (F_0_) was evaluated by the modulated measuring beam (620 nm, 5 µmol m^−2^ s^−1^), and the maximal fluorescence level of dark-adapted state (F_M_) was obtained by a 14,000 μmol m^−2^ s^−1^ saturating light pulse. The light-adapted steady-state fluorescence (F_s_) was determined in the presence of 216 μmol m^−2^ s^−1^ actinic light (635 nm) and the maximum fluorescence level (F_M_′) was determined by a saturating pulse. The minimum fluorescence level in the light-adapted state (F_0_′) was measured by illuminating the leaf with 3-s far-red light (720 nm, 5 μmol m^−2^ s^−1^) coupled with a transient absence of the actinic light. The Chl *a* fluorescence parameters were calculated as described by Poór et al. [42]: the maximal efficiency of PSII photochemistry in dark-adapted state (F_V_/F_M_); the effective quantum yield of PSII in light adapted state (Y(II)); Y(NO) and Y(NPQ), which are the fraction of energy dissipated as heat via non-regulated and regulated non-photochemical quenching processes, respectively. P700 redox changes were monitored via the difference signal of 830 and 875 nm measuring beams [43]. In analogy to F_M_ and F_M_′, the maximal p700 signal (P_M_) as well as the maximal P700 signal in light-adapted state (P_M_′) were determined, respectively. The quantum yield of PSI (Y(I)); as well as Y(ND) and Y(NA), which are the quantum yields of non-photochemical energy dissipation due to donor or acceptor side limitations of PSI, respectively, were also calculated. The extent of cyclic electron flow around PSI (Y(CEF)/Y(II)) was calculated according to Zhang et al. [44]. All calculations are summarised in Supplementary Table S1.

### 2.7. Measurement of Photosynthetic Gas Exchange Parameters

Steady state values of net CO_2_ assimilation rate (A_N_, µmol CO_2_ m^−2^ s^−1^), stomatal conductance to water vapour (g_sw_, mol H_2_O m^−2^ s^−1^), the intercellular CO_2_ concentration (C_i_) and its ratio to atmospheric (leaf chamber) CO_2_ level (C_i_/C_a_) were determined by a portable photosynthesis measuring system (LI-6400, LI-COR, Inc.; Lincoln, NE, USA) as described by Poór et al. [45]. CO_2_ was obtained from environmental source with an air flow rate of 300 µmol s^−1^, and CO_2_ concentration was maintained at 450 ppm by Soda Lime. Light was provided by LEDs emitting 635 nm (90%) and 465 nm (10%) lights with a photosynthetic photon flux density (PPFD) of 221 µmol m^−2^ s^−1^. Leaf chamber temperature was kept at 25 °C, and the relative humidity of the air at 72–75%.

### 2.8. Protein Extraction, Spectrophotometric Measurement of SOD Activity

Plant material was homogenized with double volume extraction buffer (50 mM Tris-HCl pH 7.6–7.8, containing 0.1 mM EDTA, 0.1 (*v*/*v*) % Triton X100, 10 (*v*/*v*) % glycerol) and centrifuged at 4 °C, 9300× *g* for 20 min. The supernatant was treated with 1 (*v*/*v*) % protease inhibitor cocktail (Sigma-Aldrich, St. Louis, MO, USA) and used as protein extract for further methods. To determine the protein content of plant extracts, a series of bovine serum albumin standards were used according to Bradford [46].

For SOD activity, 250 mg of plant tissues were grounded with 1 mL of extraction buffer (50 mM phosphate buffer pH 7.0 with 1 mM EDTA and 4 (*w*/*v*) % PVPP). The activity was measured *via* SOD’s ability to inhibit the reduction of NBT to formazan under light [47]. Data are shown as unit/g fresh weight, where 1 unit is equivalent to 50% inhibition of NBT reduction.

### 2.9. Native PAGE Separation of SOD and NADPH Oxidase (NOX) Isoenzymes

To visualise SOD isoenzymes, 12.5 µL protein extract (13.5 µg protein) was separated on 10 (*w*/*v*) % native polyacrylamide gel. Gels were soaked in 2.45 mM NBT for 20 min and 28 mM TEMED with 2.92 µM riboflavin for 15 min in darkness (both solutions were prepared in 50 mM phosphate buffer pH 7.8). Gels were washed with buffer two times and developed in light. To identify different isoenzymes, 2 mM KCN was used to inhibit Cu/Zn SOD isoforms and 5 mM H_2_O_2_ was used to inhibit Cu/Zn and Fe SOD isoforms, respectively. For the detection of NOX activity, 12.5 µL of protein extract (13.5 µg protein) was subjected to 10 (*w*/*v*) % native gel electrophoresis. Gels were incubated in a 10 mM Tris-HCl buffer pH 7.4, containing 0.2 mM NADPH and 0.2 mM NBT to visualise enzyme activity. DPI was used as a specific inhibitor to confirm NOX activity.

### 2.10. Western Blot Analysis of GSNOR Abundance and Protein Tyrosine Nitration

To visualise the total protein pool, denaturated leaf extracts containing 10 µg of proteins were subjected to 12 (*w*/*v*) % SDS polyacrylamide gel electrophoresis (SDS-PAGE). Gels were silver stained according to Blum et al. [48] with slight modifications. After overnight incubation in 50 (*v*/*v*) % methanol and 10 (*v*/*v*) % acetic acid containing fixation solution, gels were first soaked in sensitising and subsequently in silver containing solution. Between each step, 3 washes with distilled water were applied to clear out excess chemicals. Protein staining was developed with sodium bicarbonate containing solution, resulting in brown colour.

For western blot analysis, 7.5 µg denaturated proteins were separated in 12 (*w*/*v*) % SDS-PAGE. Proteins were transferred to a polyvinylidene fluoride (PVDF) membrane using wet blotting technique (25 mA, 16 h). Membranes were blocked in blocking solution (tris-buffered saline-Tween 20 buffer, pH 7.8) and immunolabelling started with appropriate primary antibody as below.

For protein tyrosine nitration, 1:2000 diluted 3-nitrotyrosine reactive antibodies were selected as primary antibody (produced in rabbit, Sigma-Aldrich, N0409). The secondary antibody was an anti-rabbit IgG coupled with alkaline phosphatase (produced in goat, Sigma-Aldrich, A3687) diluted at 1:10,000 concentration. The development of membranes was performed with the NBT/BCIP reaction. On all membranes, commercial nitrated bovine serum albumin (Sigma-Aldrich, N8159) was used as positive control.

To visualise the amount of GSNOR proteins, membranes were prepared as described above and as primary antibody 1:2000 diluted polyclonal antibody against GSNOR was used (produced in rabbit, Agrisera, AS09 647). The secondary antibody labelling and the development of the reaction were performed as described above.

## 3. Statistical Analysis

Results are presented as Mean ± S.E. Multiple comparison analyses were performed with SigmaStat 12 software (Systat Software, Inc., San Jose, CA, USA) using investigation of variance (ANOVA; *p* < 0.05) and Duncan’s test. Throughout the experiments, several plant generations were cultivated, and all experiments and measurements were carried out at least three times.

## 4. Results and Discussion

### 4.1. Effect of Se Doses on Shoot Growth of Stevia Seedlings

**Figure 1:**
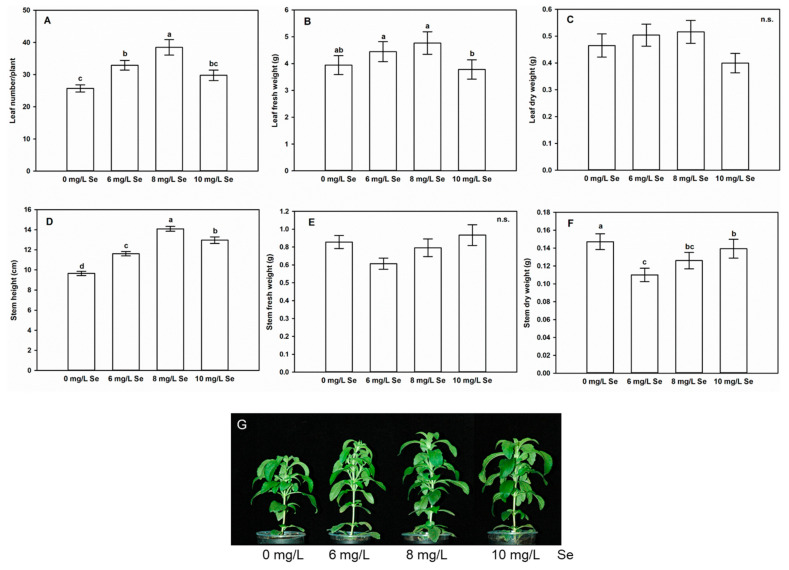
Selenium treatment affects shoot growth of *Stevia rebaudiana* L. Leaf number/plant (**A**), leaf fresh weight (g), (**B**), leaf dry weight (g), (**C**), stem height (cm), (**D**), stem fresh weight (g), (**E**) and stem dry weight (g), (**F**) of control (0 mg/L Se), 6, 8 or 10 mg/L Se-treated *Stevia*. Different letters indicate significant differences according to Duncan-test (n = 30, *p* < 0.05), “n.s.” indicates no significant difference. Representative photographs taken from control and Se-exposed Stevia shoots (**G**).

The foliar application of 6 or 8 mg/L Se significantly increased leaf number in *Stevia* seedlings (Figure 1A). Compared to untreated plants with 25 leaves, 6 mg/L Se-exposed *Stevia* developed an average of 33 leaves, while 8 mg/L Se an average of 38 leaves. These data represent 28 and 50% increase in leaf number, respectively. Interestingly, in case of the 10 mg/L Se dose, the leaf number remained at the control level. Leaf fresh weight did not change in comparison to the control in response to 6 or 8 mg/L Se but decreased due to the application of 10 mg/L Se (Figure 1B). Relative to untreated plants, no significant change in leaf dry weight caused by Se treatments was observed (Figure 1C). As for the stem, its length (shoot height) was increased by 20%, 47% or 34% as the effect of 6, 8 or 10 mg/L Se, respectively (Figure 1D,G). Se treatments did not induce significant changes in shoot fresh weight (Figure 1E), while the dry weight of the stem was decreased by all three Se treatments compared to the untreated control (Figure 1F). The largest decrease in dry weight was triggered by 6 mg/L Se, while in case of higher Se doses the reducing effect was smaller (Figure 1F).

The effect of foliar Se application shows concentration-dependence, since most of the growth parameters are positively affected by 6 or 8 mg/L Se, not influenced by 10 mg/L Se, while higher doses (e.g., 16 mg/L) reduce shoot development (data not shown). It must be noted that the concentration difference between the beneficial and the toxic Se doses is small. Plants exposed to 6 or 8 mg/L Se are taller and possess more leaves compared to control, but the biomass of the leaves is not affected by Se, and the dry weight of the stem decreases. The invariance of shoot biomass together with the increase in leaf number suggest that Se causes no overall growth induction, but a shift in biomass allocation. Intense stem elongation usually precedes flowering, thus Se-induced longitudinal growth suggests that Se may accelerate the vegetative phase and promote flowering, which is a plant strategy for stress tolerance [49]. This study did not examine *Stevia* plants at their flowering stage; although, literature data indicate that Se induces flower development [50,51]. Szarka et al. [51] treated *Stevia* plantlets with selenite or selenate at concentrations of 0, 1, 5, and 10 mg/kg through the growth medium and observed no growth induction but even growth inhibition. Their results may differ from ours due to the different mode of Se administration (here foliar spraying). At the same time, in other plant species, like Indian mustard (*Brassica juncea*), grapevine (*Vitis vinifera*), basil (*Ocimum basilicum*), tobacco (*Nicotiana tabacum*), spinach (*Spinacia oleracea*), peach (*Prunus persica*), and curly endive (*Cichorium endivia*), the beneficial effects of Se enrichment on diverse growth parameters (e.g., leaf number, plant height) have been described [52,53,54,55,56,57,58] but the applied Se doses and the ways of treatments (foliar spray, hydroponics, soil irrigation) were different. Growth stimulation by Se might be attributed to increased indole-3-acetic-acid (IAA) level and signaling, as demonstrated in tobacco shoot, where IAA concentration and expression of IAA-related genes (*YUCCA4*, *6*, *8*, *9*, *PIN1a*, *PIN1c*, *PIN4*, *PIN9*) was increased as in response to Se treatment (1 mg/L or 10 µM sodium [55,59], respectively).

The results show that Se applied at concentrations of 6–8 mg/L to leaves is beneficial for shoot growth, thus foliar Se spraying can be an effective strategy to increase the number of leaves during *Stevia* cultivation.

### 4.2. Effect of Foliar Se Application on the Total Se Content of Stevia Leaves

**Figure 2:**
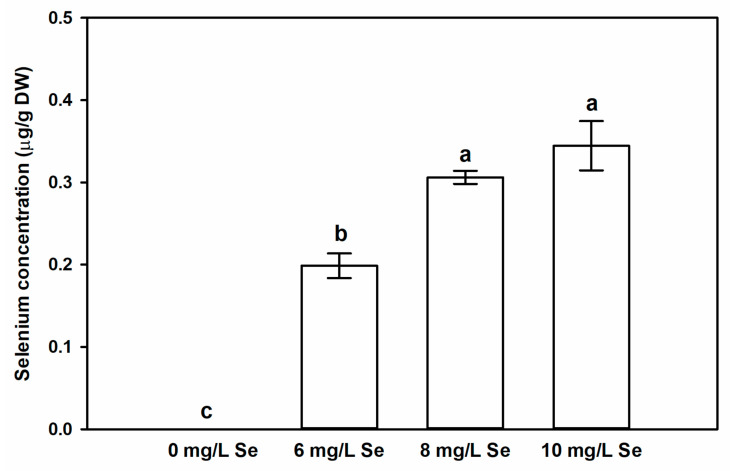
Foliar application of sodium selenate increases endogenous Se content of *Stevia* leaves. Selenium concentration (µg/g DW) in leaves of *Stevia* treated with 0, 6, 8 or 10 mg/L sodium selenate for two weeks. Different letters indicate significant differences according to Duncan-test (n = 3, *p* < 0.05).

In the leaves of untreated *Stevia* plants, the Se concentration was below the detection limit of ICP-MS. However, Se spraying resulted in a significant, concentration-dependent increase in the Se concentration of the leaves (Figure 2). The Se accumulation in the leaves might be saturated following the application of 8 mg/L Se as there was no significant difference in this respect between the 8 and 10 mg/L Se treatments, but Se accumulation was lower in the 6 mg/L Se-treated leaves. On the one hand this indicated that despite its non-selenium accumulator character, *Stevia* is able to take up and accumulate Se in its leaves and sodium selenate at the concentrations of 6, 8, or 10 mg/L can efficiently enhance endogenous Se content of *Stevia* leaves. On the other hand, the data support the view that foliar application of Se is an efficient and economical way for Se enrichment in plant tissues [60,61,62]. Notable release of Se was measured in drinks including water, coffee, and green tea sweetened with Se-fortified *Stevia* leaves [51], suggesting that Se-enriched *Stevia*-based sweetener can contribute to nutritional Se supplementation. It has to be noted that Se speciation analysis was not included in this work, but literature data suggest that, in selenate-sprayed plants, beyond inorganic Se forms of metabolized organic species such as selenomethionine, selenocysteine, or Se-methylselenocysteine may be present [63,64,65,66,67,68].

### 4.3. Effect of Se Doses on Stevioside and Rebaudioside A Content of Stevia Leaves

Stevioside and rebaudioside A were identified in *Stevia* leaf samples based on the comparison of their retention times and UV spectra with appropriate reference standards.

The concentrations of the two dominant SGs, stevioside and rebaudioside A in control and Se-treated *Stevia* leaves are shown in Table 1. In case of control plants, the concentration of stevioside was 124 ± 1.05 mg/g, while rebaudioside A content proved to be approx. a quarter of this (29.22 ± 0.27 mg/g). A slight reduction in stevioside content was observed as the effect of all three Se sprayings, and the lowest stevioside content was detected in the 8 mg/L Se-treated leaves (11% decrease compared to control). The amount of rebaudioside A also decreased by all Se concentrations, and the rate of the 8 mg/L Se-induced reduction was similar to that of stevioside (~10%). During a field experiment, integrated foliar application of Se, B, and Fe resulted in the highest total SGs content of *Stevia* leaves compared to control, while Se alone did not influence SG production significantly but improved growth parameters [69]. The synthesis of SGs via the MEP pathway is linked with photosynthetic activity and pigment production [70], thus Se-induced changes in the latter might influenced the alterations of SG content in *Stevia* leaves.

### 4.4. Effect of Selenium Doses onpigment Content of Stevia Leaves

**Figure 3:**
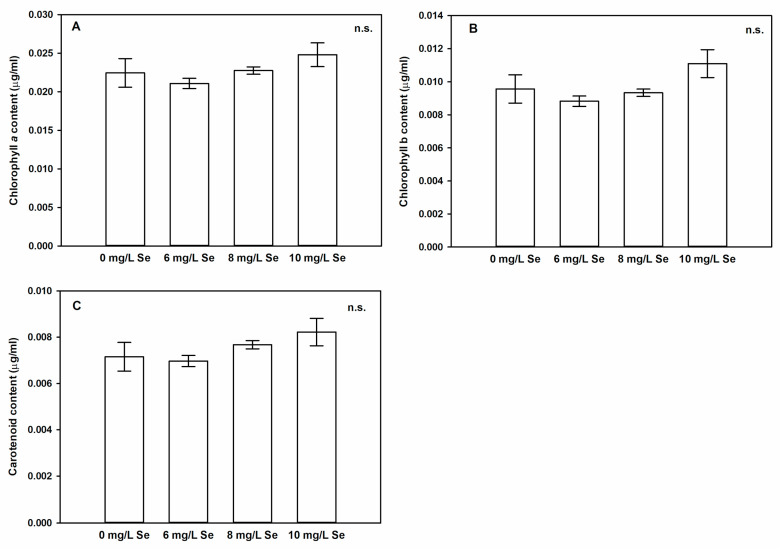
Pigment composition of *Stevia* leaves are not significantly modified by foliar Se application. Concentrations of chlorophyll a (**A**), b (**B**) and carotenoid (**C**) in leaves of *Stevia* treated with 0, 6, 8 or 10 mg/L Se. Different letters indicate significant differences according to Duncan-test (n = 8, *p* < 0.05), “n.s.” indicates no significant difference.

Although there was no visible discrepancy in leaf colour of Se-treated plants, we determined the concentrations of pigments (chl *a*, chl *b*, carotenoids) in untreated and Se-treated *Stevia* leaves. Se doses did not modify the concentrations of the main photosynthetic pigments, chl *a* and chl *b* significantly (Figure 3A,B). Similarly, the amount of carotenoids showed no statistically significant change as the effect of Se sprayings (Figure 3C). The effect of Se on leaf pigment composition was investigated by many authors, however, the alteration caused by Se seems highly species dependent [71,72,73]. The application of Se supplementation exhibited no significant effect on chl *a* and *b* or carotenoids in sugarcane [74], in oilseed rape [75], or in wheat [76] under control conditions. *Stevia* seeds primed with sodium selenite, developed seedlings with similar total chlorophyll content to control plants [77]. The lack of chlorophyll and carotenoid loss in *Stevia* leaves indicates that the applied Se concentrations were not toxic to the plants [78].

### 4.5. Effect of Selenium Doses on Photosynthetic CO_2_ Assimilation of Stevia Leaves

**Figure 4:**
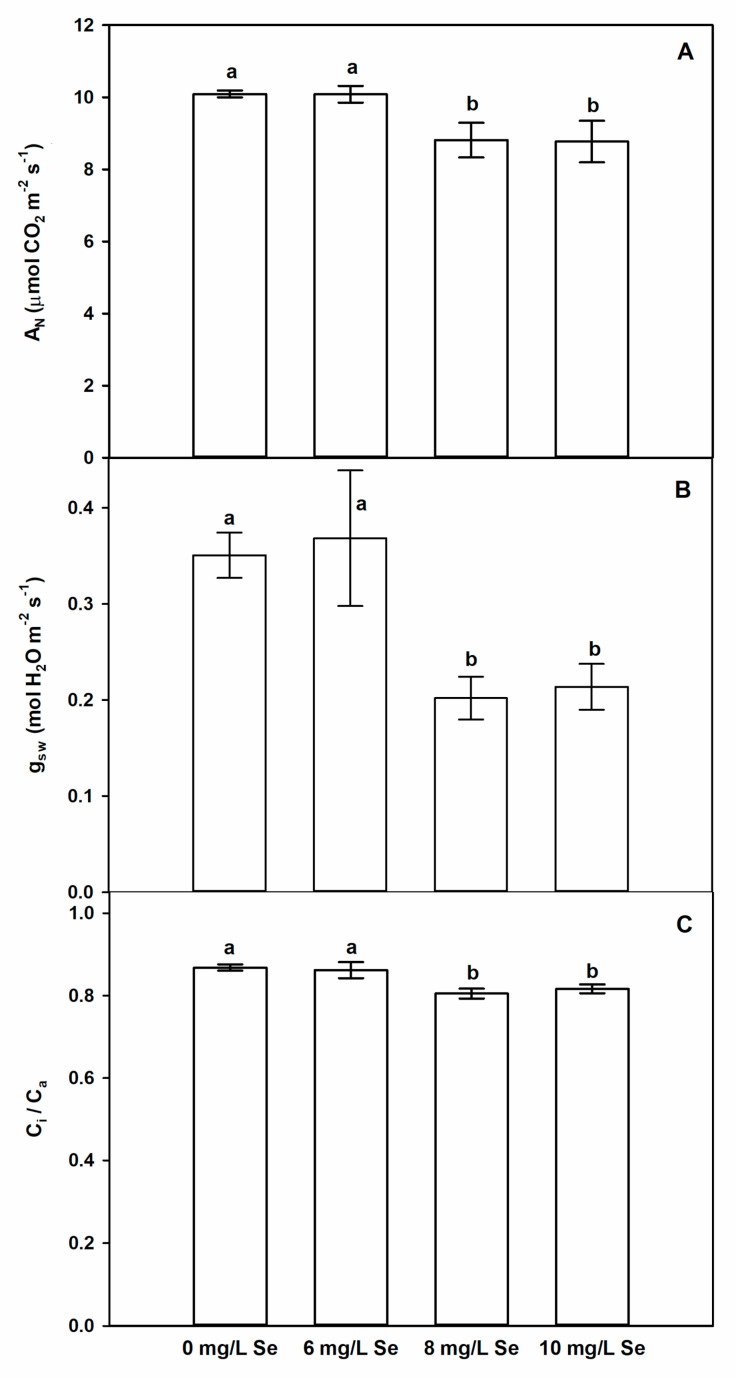
Selenium reduces CO_2_ assimilation of *Stevia* leaves due to stomatal limitation. Changes in net assimilation rate of CO_2_ (A_N,_ (**A**)), stomatal conductance to water vapour (g_sw_, (**B**)) and ratio of intercellular to atmospheric CO_2_ concentration (C_i_/C_a_, (**C**)). Different letters indicate significant differences according to Duncan-test (n = 5, *p* < 0.05).

Treating plants with 8 or 10 mg/L Se slightly, but significantly reduced net assimilation rate of CO_2_ (A_N_, Figure 4A), stomatal conductance to water vapour (g_SW_, Figure 4B) and ratio of intercellular to atmospheric CO_2_ concentration (C_i_/C_a,_ Figure 4C), which indicates that higher selenium concentrations enhanced stomatal limitation of photosynthetic CO_2_ assimilation in *Stevia* plants, but 6 mg/L Se treatments did not show significant effect on CO_2_ assimilation and related parameters. Although the effect of exogenous Se on photosynthesis has been examined in several plant species [73,79], to the best of our knowledge, there is no information available in the literature about the impact of Se supplementation on the photosynthetic activity of *Stevia* plants. Selenium supplementation mostly enhances or does not influence photosynthesis depending on the examined plant species [73]. The concentration range of Se supplementation is an important factor [80,81,82] as well as the developmental stage of the plant [83]. Although higher Se concentrations slightly limited photosynthetic CO_2_ assimilation in our system, these treatments shoved beneficial impact on leaf number or stem height. In *Brassica juncea*, Handa et al. [84] found that treatment with higher Se concentration limited photosynthetic CO_2_ assimilation and stomatal conductance in the presence of chromium, but did not limit plant growth under control circumstances.

### 4.6. Effect of Selenium Doses on Photosystem II and I Activity of Stevia Leaves

**Figure 5:**
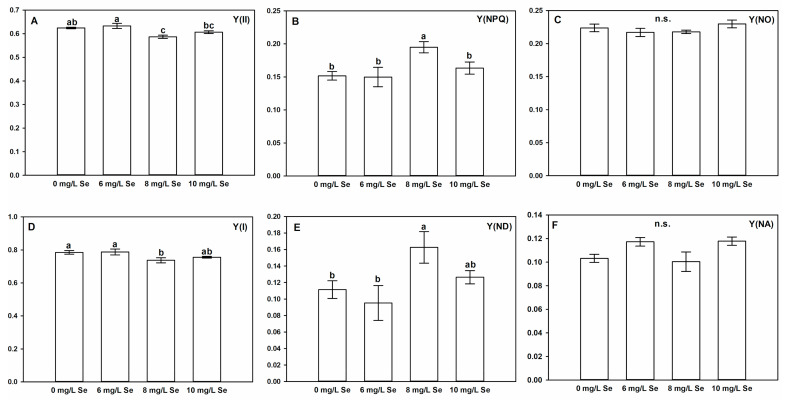
Selenium slightly alters photosynthetic activity of *Stevia* leaves. Changes in the effective quantum yield of PSII (Y(II) (**A**)), regulated (light-activated) non-photochemical energy dissipation (Y(NPQ) (**B**)), non-regulated non-photochemical energy dissipation (Y(NO) (**C**)), the quantum yield of PSI (Y(I) (**D**)), the quantum yield of non-photochemical energy dissipation due to donor side limitation of PSI (Y(ND) (**E**)) and the quantum yield of non-photochemical energy dissipation due to acceptor side limitation of PSI (Y(NA) (**F**)). Different letters indicate significant differences according to Duncan-test (n = 5, *p* < 0.05), “n.s.” indicates non-significant difference.

There were no significant changes detected in F_v_/F_m_, F_0_ (Figure S2A,B) or Y(NO) (Figure 5C) parameters, which indicates that exogenous Se caused no photodamage in PSII [85,86].

6 and 10 mg/L Se treatment did not influence PSII effective quantum yield (YII) compared to control (Figure 5A). However, 8 mg/L Se enhanced Y(NPQ) in *Stevia* leaves, which resulted in slight, but significant decrease of Y(II). Interestingly, this increase in Y(NPQ) was coupled with non-modified cyclic electron flow around PSI (Supplementary Figure S2C), which indicates that other factors might be involved in the enhancement of Y(NPQ).

Despite the fact that there are only few data in the literature about the activity and interaction of the two photosystems in *Stevia* leaves in response to Se treatments, there are examples in case of other plant species. Se application through foliar spray (10 mg/L) had no effect on Y(II) in *Glycine max*, while this concentration enhanced this parameter in *Solanum tuberosum* [73]. While the elevation of Y(NPQ) usually occurs in response to suboptimal environmental conditions as a defence mechanism [85], in our system, 8 mg/L Se treatment likely triggered a eustress-like response in PSII, as Se treatment affected PSII electron transfer processes [87].

The quantum efficiency of PSI (Y(I)) and related parameters (Figure 5D–F) followed the changes of Y(II), therefore the limitation of Y(I) caused by 8 mg/L Se treatment originated from the limitation of PSI donor side, which was also observed in wheat under Se stress [88].

### 4.7. Effect of Se Doses on SOD and NOX Activity of Stevia Leaves

**Figure 6:**
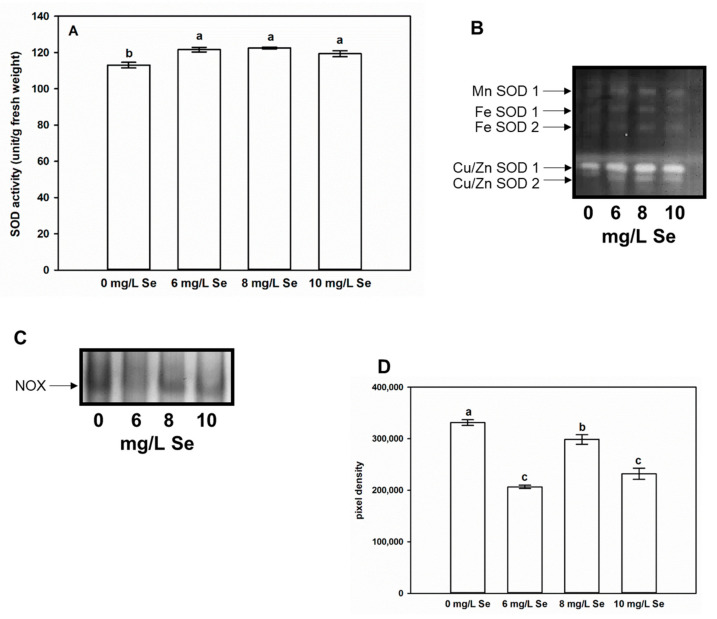
Selenium alters superoxide metabolism in *Stevia* leaves. (**A**) SOD activity (unit/g fresh weight) in leaves of *Stevia* plants treated with 0, 6, 8 or 10 mg/L Se for two weeks. Different letters indicate significant differences according to Duncan-test (n = 12, *p* < 0.05). Native PAGE separation of SOD (**B**) and NOX (**C**) isoenzymes of *Stevia* leaves. (**D**) Pixel density of the protein bands corresponding to NOX activity in *Stevia* leaves treated with 0, 6, 8 or 10 mg/L Se. Pixel densities were determined using Gelquant software (provided by biochemlabsolutions.com).

We hypothesized that low doses of Se may exert antioxidant effect, thus the activities of O_2_^●−^ scavenging and generating enzymes in *Stevia* leaves were examined. Total SOD activity was significantly increased by all three Se treatments (Figure 6A). The native gel separation of SOD isoenzymes indicated that untreated *Stevia* leaves contained two Cu/Zn SODs, two FeSODs, and one MnSOD isoenzyme (Figure 6B). In contrast to this, Moharramnejad et al. [89] detected only three SOD isoforms (SOD1, 2, and 3) in control *Stevia* leaves, and those were not identified. The observed isoenzyme pattern did not change as the effect of Se treatments. However, changes in activity were detectable. All the applied Se treatments induced the activities of both Cu/Zn SODs and the MnSOD. The measurement of protein band densities showed that all three Se treatments increased the activity of FeSOD1, while FeSOD2 activity was induced only by 6 and 8 mg/L Se compared to control (Figure 6B and Figure S2). Several reports have shown that low dosage of Se treatment increased the activity of SOD in different organs of different plant species [90,91,92,93,94,95,96]. As for isoenzymes, Se has been shown to increase the activities of all three isoenzyme types (MnSOD, FeSOD, CuSOD) in *Astragalus* species, wheat, tomato [28,83,97]. Beyond activating SOD, further putative ways of Se actions on O_2_^●−^ scavenging have been proposed including the promotion of spontaneous dismutation of O_2_^●−^ into H_2_O_2_, and the direct reaction between Se compounds and O_2_^●−^ or ^●^OH. Another theoretical mechanism of Se action may be the induction of the assembly of photosystems resulting in controlled ROS production (reviewed by Feng et al. [19]).

NADPH-containing superoxide producing NOX enzyme in *Stevia rebaudiana* has recently been characterized [98]. In agreement with this, a single specific enzyme band was detectable in native gel (Figure 6C). Its activity was significantly reduced by the application of 6 and 10 mg/L Se, while treatment with 8 mg/L Se reduced the enzyme activity to a lesser extent (Figure 6D). The involvement of NOX in Se stress-induced ROS production and toxicity has previously been reported in *Brassica rapa* [23] and in *Astragalus* species [84]. Based on this, it can be hypothesized that beneficial Se doses may cause a decrease in enzyme activity, which may be due to NO-dependent *S*-nitrosation of NOX mitigating its ability to produce O_2_^●−^ [99]. SOD activation and decreased NOX activity together may cause a downregulation of O_2_^●−^ levels in Se-treated *Stevia* leaves.

### 4.8. Effect of Selenium Doses on Nitrosative Signalling in Stevia Leaves

**Figure 7:**
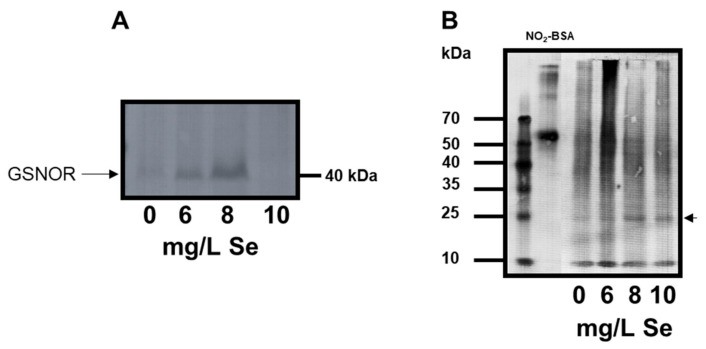
Selenium modifies nitrosative signalling in *Stevia* leaves. (**A**) Western blot probed with rabbit anti-GSNOR polyclonal antibody (1:2000) showing GSNOR protein abundance in control and Se-treated *Stevia* leaves. (**B**) Western blot probed with an antibody against 3-nitro-tyrosine (1:2000) showing nitrated proteins in *Stevia* leaves treated with 0, 6, 8 and 10 mg/L Se. Commercial nitrated BSA (NO_2_-BSA) was used as a positive control.

The amount of GSNOR protein, an enzyme that indirectly regulates protein *S*-nitrosation, was detected by western blot and found that 6 and 8 mg/L Se increased the protein amount, while 10 mg/L Se significantly decreased it compared to control (Figure 7A). Density measurement showed that the increment of GSNOR protein level was the most intense in the case of 8 mg/L Se treatment (Supplementary Figure S3). It has been reported that Se stress modifies GSNOR activity depending on the Se tolerance of *Astragalus* species [28]. In this experimental system, low Se doses-triggered increase in GSNOR abundance that could lead to downregulation of nitrosative signalling through protein *S*-nitrosation while the highest applied Se dose (10 mg/L) might exert the opposing effect.

Protein tyrosine nitration, a marker of nitrosative signalling, was detected in untreated *Stevia* leaves (Figure 7B) suggesting that a part of the protein pool of healthy *Stevia* plants is nitrated creating a physiological nitroproteome [22]. Similarly, basal tyrosine nitration was detected e.g., in sunflower, pea, pepper, *Arabidopsis thaliana*, Indian mustard grown under stress-free conditions [25,100,101,102]. The immunopositivity towards anti-3-nitrotyrosine antibody was remarkably enhanced in the 6 mg/L Se-treated *Stevia* leaf sample. Higher treatment doses (8 and 10 mg/L Se) caused control-like protein nitration, although the nitration of the protein band with 25 kDa molecular weight increased compared to the control (indicated by an arrow in Figure 7B). These indicated that low Se doses led to the intensification of protein tyrosine nitration in *Stevia* leaves. Beyond inactivating certain proteins, tyrosine nitration may influence cell signalling. Collectively, the results suggest for the first time that Se spraying intensifies nitrosative signalling in *Stevia* leaves. 

## 5. Conclusions

Based on the results, foliar Se spraying causes morphological alterations in *Stevia* shoot resulting in elongated stem and increased leaf number. However, Se cannot intensify biomass yield. Foliar Se supplementation is a suitable method for increasing the total Se content of *Stevia* leaves providing biofortification potential. Additionally, Se as an antioxidant reduces superoxide metabolism by regulating NOX and SOD enzymes thus attenuating oxidative signalling and it intensifies nitrosative signalling. Reducing oxidative and enhancing nitrosative signalling by low doses of Se may contribute to improved plant fitness. Additionally, the applied Se doses do not irreversibly and significantly inhibit photosynthesis, but Se is able to activate PSI-CEF independent protection mechanisms of the photosynthetic apparatus of *Stevia* plants.

Collectively, these results suggest that Se supplementation alters *Stevia* shoot morphology without significantly affecting biomass yield and photosynthesis but increasing Se content and performing antioxidant effects which indicate that foliar application of Se may be a promising method in *Stevia* cultivation.

## Figures and Tables

**Table 1:** Stevioside and rebaudioside A concentrations (mg/g dry weight) in leaves if *Stevia* plants were sprayed with 0, 6, 8, 10 mg/L Se twice during two weeks.

Selenium; Concentration (mg/L)	Stevioside; Concentration (mg/g)	Rebaudioside A Concentration (mg/g)
0	124.06 ± 1.05	29.22 ± 0.27
6	114.6 ± 2.82	24.16 ± 0.66
8	110.5 ± 1.10	26.24 ± 0.12
10	112.46 ± 3.20	27.2 ± 0.63

## Data Availability

Data is contained within the article or Supplementary Materials.

## Supplementary Materials

The following are available online at https://www.mdpi.com/2076-3921/10/1/72/s1, Figure S1: HPLC chromatogram of a Stevia sample (205 nm)., Figure S2: The maximal efficiency of PSII photochemistry in dark-adapted state (Fv/Fm; A), The minimal fluorescence level in dark adapted leaves (F0; B), The extent of cyclic electron flow around PSI (Y(CEF)/Y(II); C)., Figure S3: Pixel density of the protein bands corresponding to GSNOR protein abundance in Stevia leaves treated with 0, 6, 8 or 10 mg/L Se. Table S1: Calculated parameters of Chl a fluorescence and PSI activity.
