# Supplementary material for: The Effect of Foliar Selenium (Se) Treatment on Growth, Photosynthesis, and Oxidative-Nitrosative Signalling of Stevia rebaudiana Leaves
Source: Antioxidants (Basel). 2021 Jan 8;10(1):72. doi: 10.3390/antiox10010072 (PMC7826996; doi:10.3390/antiox10010072)
Supplement: Supplementary file 1 [file antioxidants-10-00072-s001.pdf]

Table S1. Calculated parameters of Chl *a* fluorescence and PSI activity [43] Klughammer and Schreiber 1994, [44] Zhang et al. (2014a), [45] Poór et al. 2019b).

F_V/F_M	$F_V/F_M = (F_M - F_0) / F_M$
Y(II)	$Y(II) = (F_M' - F_s) / F_M'$
Y(NPQ)	$Y(NPQ) = 1 - Y(II) - 1/ ((F_M / F_M' - 1) + 1 + [(F_M' - F_s) / (F_M' - F_0') \times F_0' / F_s] \times (F_M / F_0 - 1))$
Y(NO)	$Y(NO) = 1 / ((F_M / F_M' - 1) + 1 + [(F_M' - F_s) / (F_M' - F_0') \times F_0' / F_s] \times (F_M / F_0 - 1))$
Y(I)	Y(I) = 1 - Y(ND) - Y(NA)
Y(ND)	Y(ND) = 1 – P700red
Y(NA)	$Y(NA) = (P_M - P_M') / P_M$
Y(CEF)/Y(II)	Y(CEF)/Y(II) = [Y(I) – Y(II)] / Y(II)

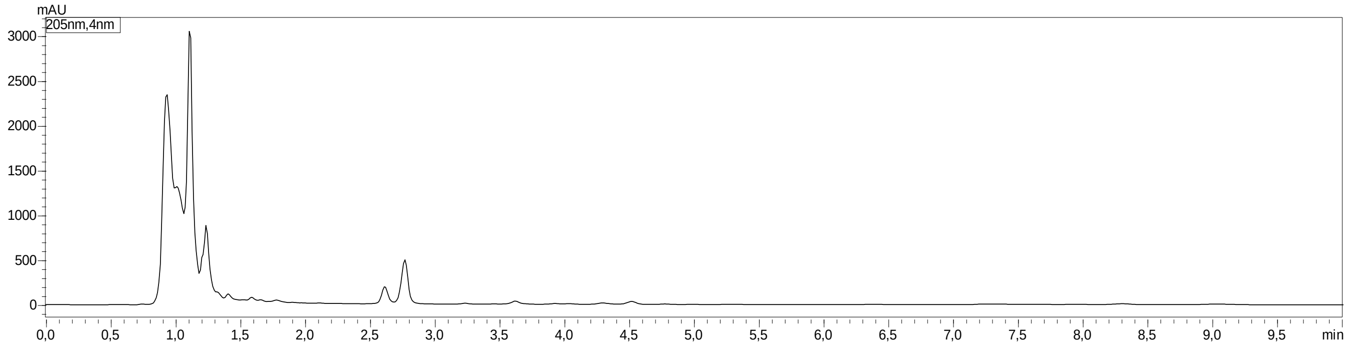

Figure S1. HPLC chromatogram of a *Stevia* sample (205 nm). Rebaudioside A was eluted at 2.6 min, whereas stevioside at 2.8 min. Since the analysed steviol glycosides are characterized by UV absorption maxima at 205 nm, quantification was carried out at this wavelength. The calibration curves of the reference standards were established based on 7 points (R^2 for rebaudioside is 0.9939239, and 0.9945832 for stevioside, respectively). The analytical method was characterized by limit of detection (LOD) values of 2,123 µg/inject and 11,764 µg/inject for rebaudioside A and stevioside, respectively, whereas the limit of quantification values were 6433 µg/inject and 35,649 µg/inject for these two analytes.

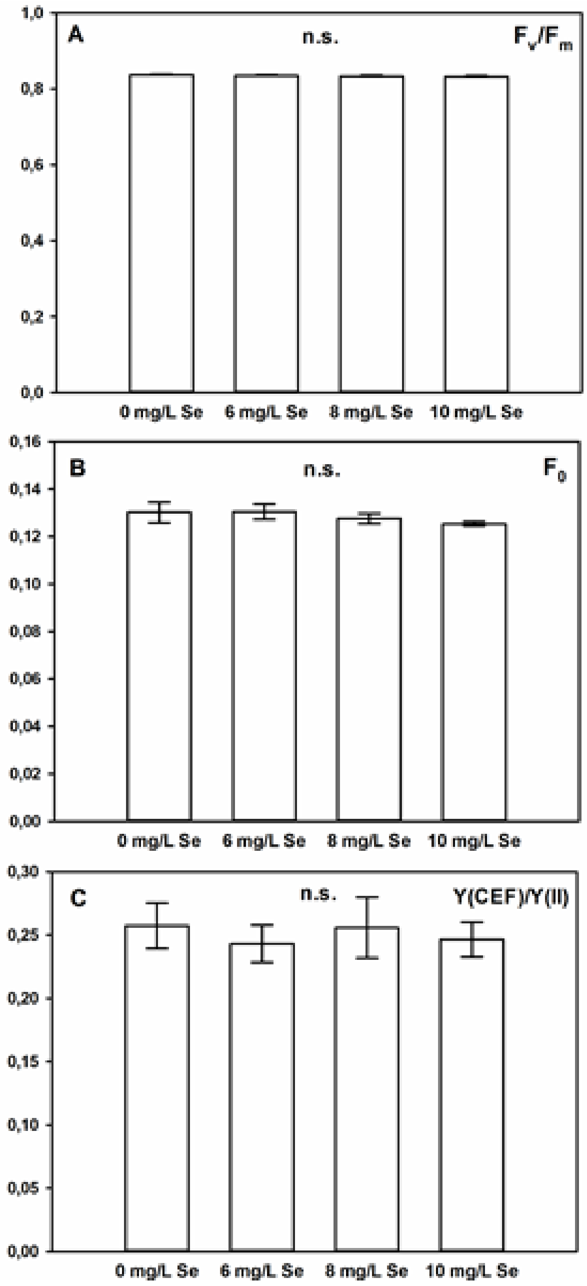

Figure S2. The maximal efficiency of PSII photochemistry in dark-adapted state (F_v/F_m) (**A**), The minimal fluorescence level in dark adapted leaves (F_0), (**B**), The extent of cyclic electron flow around PSI (Y(CEF)/Y(II); (**C**). „n.s." indicates non-significant difference.

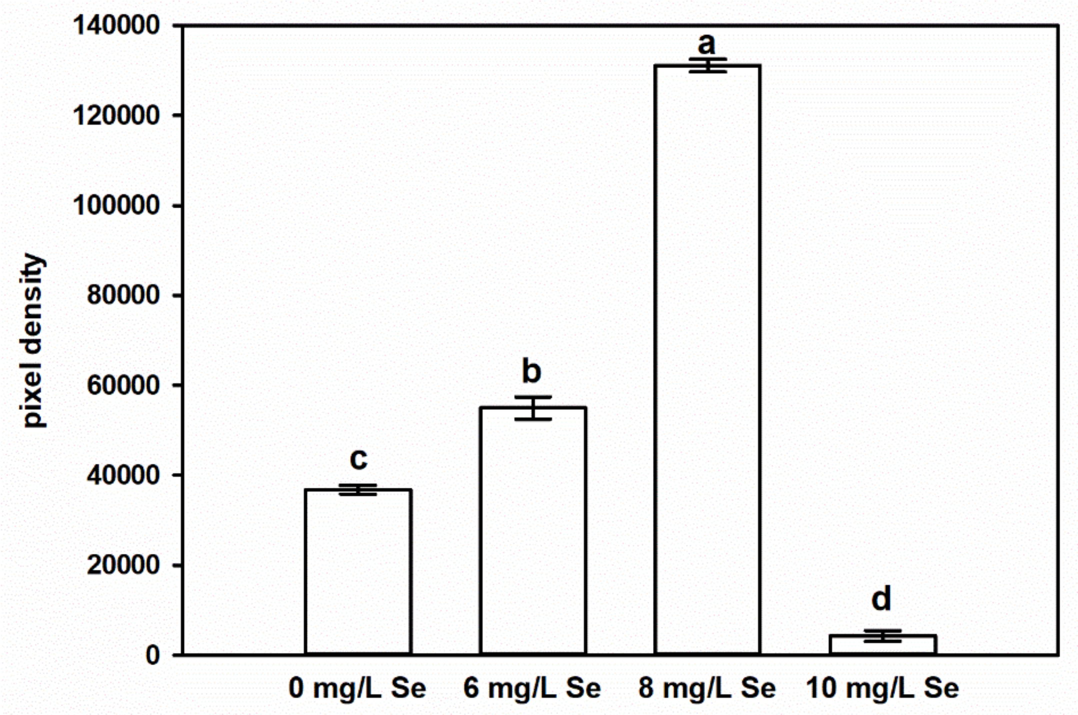

Figure S3. Pixel density of the protein bands corresponding to GSNOR protein abundance in *Stevia* leaves treated with 0, 6, 8 or 10 mg/L Se. Pixel densities were determined using Gelquant software (provided by biochemlabsolutions.com).
